# A computational model of prefrontal cortex based on physiologically derived cellular parameter distributions

**DOI:** 10.1186/1471-2202-14-S1-P116

**Published:** 2013-07-08

**Authors:** Joachim Hass, Loreen Hertäg, Sebastian Claudio Quiroga Lombard, Tatiana Golovko, Daniel Durstewitz

**Affiliations:** 1 Bernstein-Center for Computational Neuroscience, Central Institute of Mental Health, Medical Faculty Mannheim, Heidelberg University, Mannheim, 68159, Germany

## 

A major theme in computational neuroscience is to derive neuronal network models that are on the one hand side physiologically realistic, thus allowing reasonable inferences on their biological counterparts, yet on the other hand are still computationally tractable in some sense (e.g., in terms of simulation time requirements).

Towards this goal, we constructed a computational network model of the PFC based on simple single neuron elements, yet equipped with highly realistic anatomy and close fitting of cellular and synaptic parameter distributions derived from an extensive data base of prefrontal in vitro and in vivo recordings. The resulting model was reasonably fast to simulate and fit to data, amenable to mean-field approaches, yet exhibiting a high degree of physiological validity, even in a predictive sense. At the single-cell level, the network is based on the adaptive exponential integrate-and-fire model (AdEx, [1]) which we simplified further (simpAdEx [2]) to yield closed-form expressions for the firing rates under transient and steady-state conditions. These are used for fully automatized fitting to f-I and V-I curves of real neurons, with fitting times 1-2 orders of magnitude faster than for the full AdEx, thus allowing relatively quick adaptation to large data bases. The resulting model is also able to predict spiking times of neurons driven by in-vivo-like fluctuating currents that were not used for fitting [2]. In vitro recordings from ~200 L2/3 and L5 pyramidal cells, fast-spiking and bitufted interneurons from adult rodent PFC were used to generate a distribution of model cells that reflects the diversity of neurons in the real PFC. At the synaptic level, data obtained from short-term plasticity protocols were used to fit models of synaptic dynamics, with parameter distributions matched to the types of the connected neurons [3]. At the anatomical level, the network structure involves stripe-like columns as observed in primate PFC [4], a laminar organization and clustered connectivity [5], where the connectivity distributions are extracted from an extensive literature research.

Even without any tuning, the network model already manages to approximate a number of statistics extracted from in-vivo data from awake behaving rats [6]. We also used these in-vivo data sets for further tuning of synaptic parameters. It turned out that a network dynamic with in-vivo-like properties depended sensitively on the matching of cellular model parameter distributions to the true multivariate distributions obtained empirically, i.e. taking both marginal distributions as well as at least their pairwise covariations into account. In summary, we have generated a PFC network model which is biologically highly valid, yet still reasonably fast to simulate and fit, and can now be exploited to probe the impact of pharmacological or genetic conditions on network dynamics, and to model performance in PFC-dependent tasks.
